# Electrochemical Analysis of Antichemotherapeutic Drug Zanosar in Pharmaceutical and Biological Samples by Differential Pulse Polarography

**DOI:** 10.1155/2013/420761

**Published:** 2013-12-24

**Authors:** Chennupalle Nageswara Reddy, Puthalapattu ReddyPrasad, NeelamYughandhar Sreedhar

**Affiliations:** ^1^Department of Chemistry, Government Degree College, Kodur, Kadapa District, Andhra Pradesh, India; ^2^Graduate Institute of Applied Science and Technology, National Taiwan University of Science and Technology, Taipei 10607, Taiwan; ^3^Electroanalytical Lab, Department of Chemistry, Sri Venkateswara University, Tirupati, Andhra Pradesh, India

## Abstract

The electrochemical reduction of zanosar was investigated systematically by direct current polarography, cyclic voltammetry, and differential pulse polarography (DPP). A simple DPP technique was proposed for the direct quantitative determination of anticancer drug zanosar in pharmaceutical formulation and spiked human urine samples for the first time. The reduction potential was −0.28 V versus Ag/AgCl with a hanging mercury drop electrode in Britton-Robinson buffer as supporting electrolyte. The dependence of the intensities of currents and potentials on pH, concentration, scan rate, deposition time, and nature of the supporting electrolyte was investigated. The calibration curve was found to be linear with the following equation: *y* = 0.4041*x* + 0.012, with a correlation coefficient of 0.992 (*R* ^2^) over a concentration range from 1.0 × 10^−7^ M to 1.0 × 10^−3^ M. In the present investigation, the achieved limit of detection (LOD) and limit of quantization (LQD) were 7.42 × 10^−8^ M and 2.47 × 10^−8^ M; respectively. Excipients did not interfere with the determination of zanosar in pharmaceutical formulation and spiked urine samples. Precision and accuracy of the developed method were checked by recovery studies in pharmaceutical formulation and spiked human urine samples.

## 1. Introduction

Zanosar (common name streptozotocin) is a chemotherapy agent in the nitrosourea class. It is an anticancer chemotherapy drug and naturally occurring chemical which is particularly toxic to the insulin-producing beta cells of the pancreas in mammals. In particular, it is used in medicine for treating certain cancers of the islets of Langerhans and used in medical research to produce an animal model for Type 1 diabetes in large dose as well as Type 2 diabetes in multiple low doses. Streptozotocin was originally identified in the late 1950s as an antibiotic [1]. In the mid- of 1960s, it was found to be selectively toxic to the beta cells of the pancreatic islets, the cells which normally regulate blood glucose levels by producing the hormone insulin. It suggested the drug's use as an animal model of diabetes [2, 3] and as a medical treatment for cancers of the beta cells [4]. However, the National Cancer Institute investigated the use of streptozotocin in cancer chemotherapy in the 1960s and the 1970s.

Streptozotocin was approved by the US Food and Drug Administration (FDA) for treating metastatic cancer of the pancreatic islet cells. Since it carries a substantial risk of toxicity and rarely cures the cancer, its use is generally limited to patients whose cancer cannot be removed by surgery. In these patients, streptozotocin can reduce the tumour size and reduce symptoms [5]. A typical dose is 500 mg/m^2^/day by intravenous injection for 5 days, repeated every 4–6 weeks. Owing to its high toxicity to beta cells, in scientific studies, streptozotocin has also been long used for inducing insulitis and diabetes in experimental animals [6, 7].

A review of the literature reveals that most of the methods are used for analysis of streptozotocin by HPLC [8–13]. In the recent years, the electrochemical techniques have led to the advancement in the field of analysis because of their sensitivity, low cost, and relatively short analysis time when compared with other techniques. However, additional application of electroanalytical techniques includes the determination of reaction mechanisms. Redox properties of a drug can give insights into it's metabolic fate or its *in vivo* redox processes or pharmaceutical activity [14–18]. Nevertheless, the critical literature survey revealed that no attempt has been made to investigate the electrochemical behaviour of the zanosar in pharmaceutical formulation and human urine samples. Therefore, this work attempted to establish experimental conditions for the study of the electrochemical behavior of zanosar by direct current polarography, cyclic voltammetry, differential pulse polarography, controlled potential electrolysis and millicoulometry. Another the aim of the present work is to develop and validate a rapid, simple, selective differential pulse polarography method for the determination of zanosar in pharmaceutical formulation and human urine samples.

## 2. Experimental

### 2.1. Apparatus and Reagents

The measurements were performed by an Elico Model CL-362 polarographic analyzer, a conventional three-electrode system consisting of an HMDE (area 0.02323 cm^2^) as the working electrode, an Ag/AgCl reference electrode and platinum counter electrode was used in all experiments. It was outfitted with a Model EPSON LX-300^+^ *X*-*Y* recorder. Cyclic voltammetric measurements were carried on Metrohm 757 VA Computrace Model AFRDE4 potentiostat and MSRX speed control unit coupled with digital electronics 200*X*-*Y*/*t* recorder. The effect of pH on the half-wave potentials and diffusion-limited current for zanosar at a concentration of 1.0 × 10^−5 ^M was studied over the pH range of 2.0–12.0. The corresponding voltammograms were recorded under identical conditions and all the experiments were performed at an ambient temperature (25 ± 1°C). The limit of detection (LOD) was calculated by using equation dl = 3(SD/*m*), where dl = detection limit, SD = standard deviation, and *m* = slope of the calibration plot.

All chemicals were analytical reagent grade chemicals and doubly distilled water was used in preparation of all solutions. Stock solution (1.0 × 10^−3 ^M) was prepared by dissolving zanosar in dimethylformamide (DMF). All dilute solutions were freshly prepared daily from the stock solution. Britton-Robinson (BR) buffer solutions ranging from pH 2.0 to 12.0 were prepared using 0.2 M, 0.01 M citric acid and 0.1 M trisodium orthophosphate.

### 2.2. Recommended Procedure

The recommended solution of zanosar (1.0 × 10^−5^ M) was prepared by dilution of stock solution with a suitable amount of DMF. 1.0 mL of the standard solution was transferred into the voltammetric cell and made up 9.0 mL with the supporting electrolyte for achieving the required concentration and then deoxygenated by bubbling oxygen free nitrogen gas for 10 min. After recording the voltammogram, small increments of the standard solutions are added and then voltammograms were recorded for each addition under similar conditions. The optimum conditions for the analytical determination of zanosar at pH 4.0 were found to be a drop time of 2 sec, pulse amplitude of 40 mV and reduction potential of −0.28 V versus saturated Ag/AgCl electrode.

## 3. Results and Discussion

### 3.1. DC Polarography

**Figure 1:**
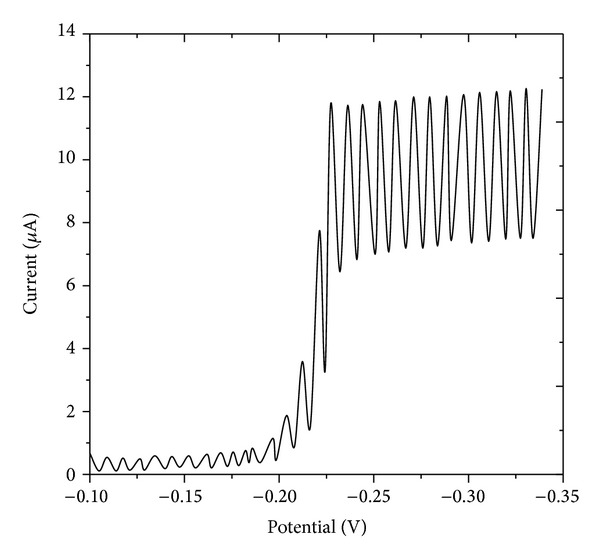
Typical DC polarogram of zanosar at pH 4.0, concentration: 1.0 × 10^−5^ M, and drop time: 2 sec.

The well-defined reduction peak has been observed for the zanosar in BR buffer (pH ranging from 2.0 to 12.0). A typical DC polarogram was presented in Figure 1. From Tomes' criterion, log-plot analysis, and dependence of half-wave potentials on drop time, the reduction process has been identified to be irreversible. The obtained linear plots of *i*_*d*_ versus *h* ^1/2^ passing through the origin in the supporting electrolyte indicate that the reduction process was adsorption free in the BR buffer supporting electrolyte at the pH 4.0, where weak adsorption of the reactant was observed [18]. The results observed have been used for evaluation of kinetic parameters such as diffusion coefficient, transfer coefficient and heterogeneous forward rate constants for the reduction process of >C=O group which have been reported in Table 1.

### 3.2. Cyclic Voltammetry

**Figure 2:**
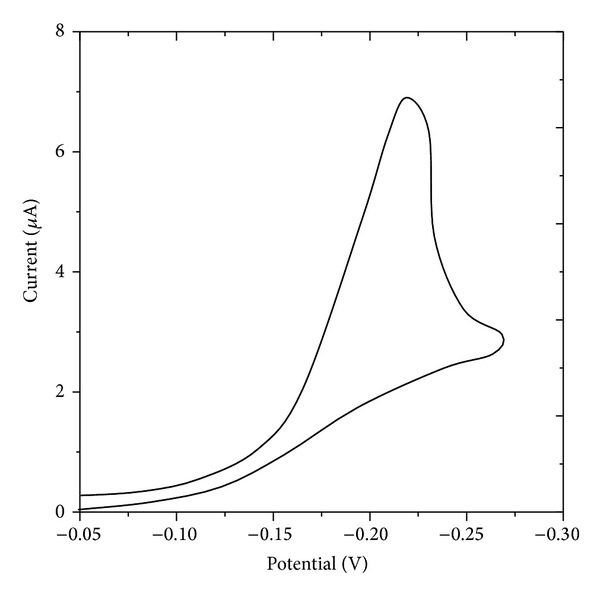
Cyclic voltammogram of zanosar at HMDE, scan rate: 40 mVs^−1^, concentration: 1.0 × 10^−5^ M, and pulse amplitude: 40 mV.

The linear plot of *I*_*p*_ versus *v* ^1/2^ passing through origin indicates the electrode process to be mainly diffusion controlled adsorption on the electrode surface in all the pH ranges studied [18]. The peak current (*I*_*p*_) was dependent on scan rate (*v*) and pH of the supporting electrolyte. The reduction process was found to be irreversible and presence of anodic peak on the reverse scans in cyclic voltammetry as well as evidence of Tomes' criterion confirms the electrode process to be reversible. One proton has participated in the rate determining step for the reduction of >C=O group. The typical cyclic voltammogram is shown in Figure 2 and cyclicevoltammtric data of zanosar has been reported in Table 2.

### 3.3. Differential Pulse Polarography

**Figure 3:**
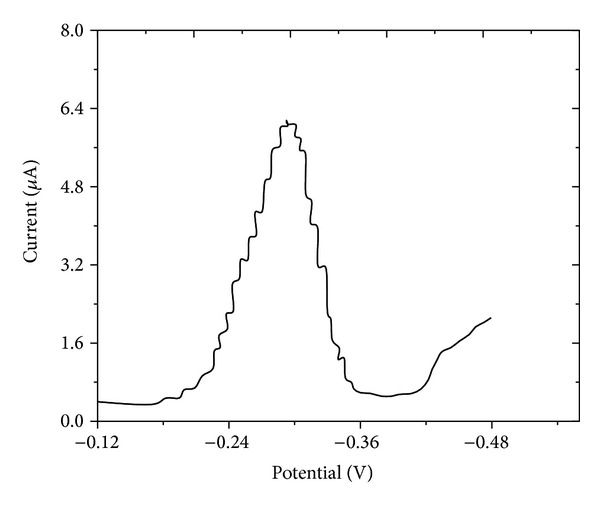
Typical differential pulse polarogram of zanosar at pH 4.0, concentration: 1.0 × 10^−5^ M; pulse amplitude, and 40 mV, drop time: 2 sec.

**Figure 4:**
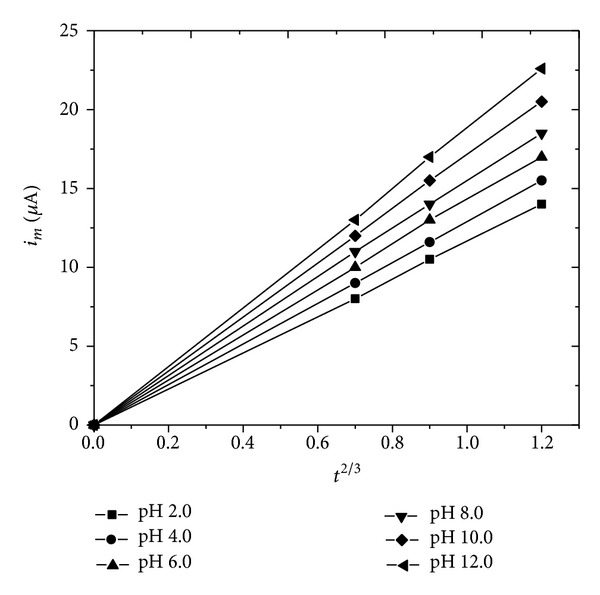
*i*_*m*_ versus *t* ^2/3^ plots of zanosar.

The differential pulse polarography measurements have been carried out in the present investigation for the analysis of zanosar in pharmaceutical formulation and spiked human urine samples with supporting electrolyte of BR buffer as shown in Figure 3. No more than one well defined peak has been observed in the supporting electrolyte at pH 4.0. The variation of peak potential with concentration indicates the irreversible nature of the electrode process. The plots of *i*_*m*_ versus *t* ^2/3^ (Figure 4) being linear and not passing through origin in all the pH ranges, show that adsorption complication was involved. Kinetic parameters have been evaluated and presented in Table 3.

### 3.4. Kinetic Data and Electrode Mechanism

**Figure 5:**
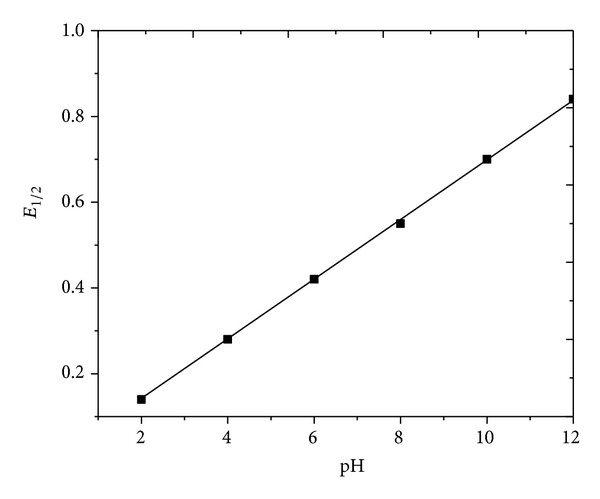
*E*_1/2_ versus pH plots of zanosar.

**Figure 6:**
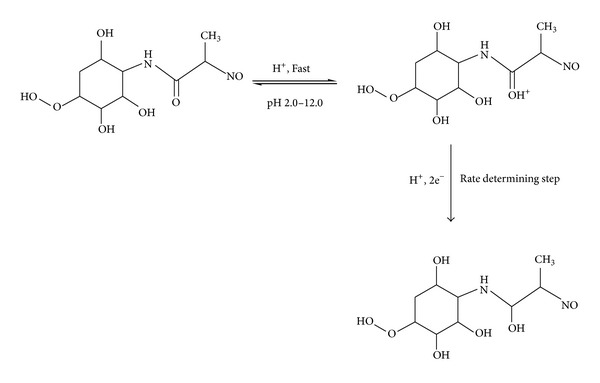
Electrochemical mechanism of zanosar.

Under the experimental conditions described above, the polarographic reduction of zanosar gave a single well-defined two-electron reduction peak at pH 2.0–12.0 with all the techniques, corresponding to the reduction of carbonyl groups. The diffusion coefficient values have been seen to gradually decrease which account for the decrease in diffusion current with an increase in pH due to the less availability of protons. The heterogeneous forward rate constant values of the compound under investigation have been seen to gradually decrease with an increase in pH of the supporting electrolyte. It may account for the shift of reduction potentials towards more negative values with an increase in pH. The plot of *E*_1/2_ versus pH has been shown in Figure 5. The *E*_1/2_ versus pH plot and using the number of protons participated in the rate determining step of >C=O group reduction are shown in Figure 6. This peak was attributed to the reduction of >C=O group of which one was at position involving one-proton and two-electrons which are the most useful for analytical purposes. An increase in pH increases the dissociation constant of the protonated species and these factors affect the protonation rate and consequently the reduction potentials are shifted to more negative values. On the basis of the experimental results obtained, the mechanism could be suggested for the voltammetric reduction of zanosar, which corresponds to the usual reduction mechanism for the >C=O group.

The total number of electrons involved during the electrode reduction of zanosar was determined by millicoulometry [19]. The number of protons and electrons involved in the overall reduction process of zanosar was found to be two and four, respectively. Controlled potential electrolysis experiments were conducted at −0.28 V versus Ag/AgCl electrode at pH 4.0 and the corresponding decay was noted using a galvanometer. The electrolysis was allowed to proceed virtually to completion. The electrolyzed sample was dried and the product was separetad from supporting electrolyte by extraction with diethyl ether; the product was identified as the hydroxy derivative, and identity was confirmed by infrared spectral studies in which the characteristic peak of the hydroxyl derivative was observed in the frequency range of 3550–3350 cm^−1^.

### 3.5. Applications

Zanosar in pharmaceutical formulation namely streptozotocin should be administered intravenously by rapid injection or short/prolonged infusion. It's not active orally containing a 500 mg/m^2^ of compound in a total injection mass of approximately 1000 mg/m^2^ which has been analysed in order to examine the applicability of the method. A portion equivalent amount of the compound was weighed accurately and dissolved in dimethylformamide and transferred into 10 mL calibrated flasks where it was described in the general procedure. With the help of calibrated graph, the amount of zanosar in portion of the sample was calculated. From the recovery studies; formulations containing zanosar at pH 4.0 are given in Table 4. The obtained recoveries are in the range from 99.40% to 99.93% for streptozotocin samples with relative standard deviation 0.22–0.38% for five replicates.

The applicability of the method for the estimation of zanosar in urine was checked by using different spiked human urine samples coupled with a standard addition method. Direct determinations of undigested urine may yield for certain probands unreliable data due to trace metal trapping by natural chelating substances. An aliquot of 2.0 mL of urine without treatment was diluted with water to 25 mL in a volumetric flask and 0.1 mL aliquot of this solution was transferred into a 25 mL calibrated flask; 6 mL or BR buffer (pH: 4.0) was added and diluted to the mark with water. The samples were measured according to the above described procedure. The voltammograms of samples without zanosar do not show any signal which can interfere with direct determination. The urine samples were used not only to study various possibilities to overcome a biomatrix effect and to find the best solution but also to explore the way for simple, reliable, fast determination of zanosar in urine by its dilution on employing differential pulse polarography. The potential interference of some urine ingredients both organic and inorganic salts was checked without adding depolarizer. None of these ingredients did affect the determination of zanosar. The recoveries are in the range from 99.00% to 99.73% with relative standard deviation 0.09–0.50% for five replicates. The results were presented in Table 5. The described method was less time consuming than other methods and has satisfactory precision.

### 3.6. Calibration Plot

**Figure 7:**
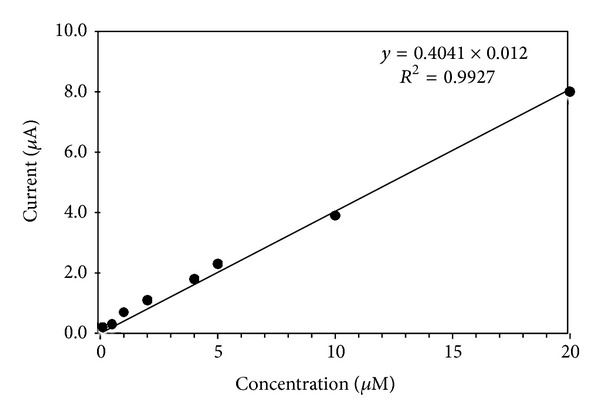
Calibration curve for determination of zanosar with proposed method.

The Differential pulse polarography well-defined wave/peak obtained at pH 4.0 is used in the analytical estimation of zanosar. Both standard addition and calibration methods are used for the analysis of zanosar in pharmaceutical formulations and urine samples. The peak current is found to vary linearly with the concentration of zanosar over the range from 1.0 × 10^−7^ M to 1.0 × 10^−3^ M with the lower detection limit of 7.42 × 10^−8^ M (Figure 7). The calibration curve has been found to be linear with the equation *y* = 0.4041*x* + 0.012, with a correlation coefficient of 0.992 (*R* ^2^), and relative standard deviation values are found to be 0.315%, respectively, for five replicates. The validation results were summarised in Table 6.

## 4. Conclusion

The electrochemical behaviour of zanosar at hanging mercury drop electrode was examined in BR buffer over a pH range from 2.0 to 12.0 by D.C polarography, differential pulse polarography, and cyclic voltammetric methods. The diffusion coefficient, transfer coefficient and heterogeneous forward rate constant values were noticed to be in good agreement in all the techniques. A fully validated, simple, sensitive, selective, fast and low-cost differential pulse polarography methods were developed for the determination of zanosar in pharmaceutical formulations and in spiked human urine samples. The described method could be recommended for use in trace analysis, quality control, and clinical laboratories.

## Figures and Tables

**Table 1:** Typical DC polarographic data of zanosar (concentration: 1.0 × 10^−5^ M).

pH of the supporting electrolyte	−*E*_1/2_/V	*i*_*d*_/*μ*A	*α*_na_	*D* × 10^6^/cm^2^ s^−1^	*K* ^*o*^ *f*, *h*/cm s^−1^
2.0	0.15	5.80	0.76	2.02	2.04 × 10^−14^
4.0	0.24	5.42	0.75	2.14	2.64 × 10^−13^
6.0	0.39	5.05	0.73	2.98	3.14 × 10^−13^
8.0	0.42	4.73	0.68	2.84	3.02 × 10^−14^
10.0	0.60	4.62	0.69	1.86	3.26 × 10^−14^
12.0	0.68	4.55	0.62	1.76	3.82 × 10^−14^

**Table 2:** Typical cyclic voltammetric data of zanosar (concentration: 1.0 × 10^−5^ M).

pH of the supporting electrolyte	−*E*_*p*_/V	*i*_*p*_/*μ*A	*α*_na_	*D* × 10^6^/cm^2^ s^−1^	*K* ^*o*^ *f*, *h*/cm s^−1^
2.0	0.16	5.65	0.85	3.18	2.06 × 10^−14^
4.0	0.29	5.88	0.89	3.06	2.19 × 10^−13^
6.0	0.35	6.15	0.93	2.98	3.62 × 10^−14^
8.0	0.48	6.40	0.82	2.86	3.64 × 10^−14^
10.0	0.57	6.25	0.96	2.56	3.04 × 10^−13^
12.0	0.72	6.35	0.84	2.32	3.14 × 10^−14^

**Table 3:** Typical differential pulse polarographic data of zanosar (concentration: 1.0 × 10^−5^ M).

pH of the supporting electrolyte	−*E*_*m*_/V	*i*_*m*_/*μ*A	*α*_na_	*D* × 10^6^/cm^2^ s^−1^	*K* ^*o*^ *f*, *h*/cm s^−1^
2.0	0.17	4.98	0.96	2.41	4.60 × 10^−14^
4.0	0.29	4.84	0.95	2.28	4.21 × 10^−13^
6.0	0.36	4.62	0.93	2.11	3.80 × 10^−13^
8.0	0.49	4.52	0.88	1.99	4.04 × 10^−13^
10.0	0.53	4.49	0.87	1.89	3.97 × 10^−13^
12.0	0.69	4.35	0.84	1.74	4.18 × 10^−14^

**Table 4:** Polarography assay of zanosar in pharmaceutical formulations by differential pulse polarography.

Name of the formulation	Labelled amount	Amount found*	Recovery (%)	Standard deviation	% RSD
Streptozotocin	5.0	4.97	99.40	0.017	0.34
	10.0	9.98	99.80	0.022	0.22
	15.0	14.99	99.93	0.490	0.38

*n = 5 (*n*: no of replicates).

**Table 5:** Polarography assay of zanosar in spiked urine samples by differential pulse polarography.

Sample	Labelled amount	Amount found*	Recovery (%)	Standard deviation	% RSD
1	5.0	4.95	99.00	0.018	0.36
2	10.0	9.92	99.20	0.050	0.50
3	15.0	14.96	99.73	0.014	0.09

*n = 5 (*n*: no of replicates).

**Table 6:** Analytical parameters for the developed method.

Parameters	DPP method
Working electrode	HMDE
pH	4.0
Linearity range (M)	1.0 × 10^−7^ M to 1.0 × 10^−3^
Slope	0.4041
Intercept	0.012
Correlation coefficient	0.9927
LOD (M)	7.42 × 10^−8^
LQD (M)	2.47 × 10^−8^
RSD (%)	0.315
